# Synthetic PreImplantation Factor (sPIF) induces posttranslational protein modification and reverses paralysis in EAE mice

**DOI:** 10.1038/s41598-019-48473-x

**Published:** 2019-10-02

**Authors:** Soren Hayrabedyan, Reut Shainer, Zhanna Yekhtin, Lola Weiss, Osnat Almogi-Hazan, Reuven Or, Charles L. Farnsworth, Scott Newsome, Krassimira Todorova, Michael J. Paidas, Chaya Brodie, Eytan R. Barnea, Martin Mueller

**Affiliations:** 1 Institute of Biology and Immunology of Reproduction, Bulgarian Academy of Sciences, Laboratory of Reproductive OMICs Technologies, Sofia, Bulgaria; 2 Department of Bone Marrow Transplantation and Cancer Immunotherapy, Hadassah Hebrew University Medical Center, Jerusalem, Israel; 3 Molecular Biology of Bones and Teeth Section, National Institute of Dental and Craniofacial Research, National Institutes of Health, Bethesda, MD USA; 4 Cell Signaling Technology, INC., Danvers, MA USA; 5 Division of Neuroimmunology and Neuroinfectious Diseases, Johns Hopkins Hospital, Baltimore, MD USA; 6 Department of Obstetrics, Gynecology and Reproductive Sciences, Yale University School of Medicine, New Haven, CT USA; 7 Department of Obstetrics, Gynecology Reproductive Sciences, University of Miami Miller School of Medicine, Miami, Florida USA; 8 The Mina and Everard Goodman Faculty of Life Sciences, Bar-Ilan University, Ramat-Gan, Israel; 9 Davidson Laboratory of Cell Signaling and Tumorigenesis, Hermelin Brain Tumor Center, Department of Neurosurgery, Detroit, MI USA; 10 Society for the Investigation of Early Pregnancy (SIEP), New York, NY 10016 USA; 11 BioIncept, LLC, New York, NY 10016 USA; 12 Department of Obstetrics and Gynecology, University Hospital Bern, Friedbuehlstrasse 19, 3010 Bern, Switzerland; 13 Department of Paediatrics, Maastricht University, P. Debyelaan 25, 6229 Maastricht, The Netherlands

**Keywords:** Post-translational modifications, Multiple sclerosis

## Abstract

An autoimmune response against myelin protein is considered one of the key pathogenic processes that initiates multiple sclerosis (MS). The currently available MS disease modifying therapies have demonstrated to reduce the frequency of inflammatory attacks. However, they appear limited in preventing disease progression and neurodegeneration. Hence, novel therapeutic approaches targeting both inflammation and neuroregeneration are urgently needed. A new pregnancy derived synthetic peptide, synthetic PreImplantation Factor (sPIF), crosses the blood-brain barrier and prevents neuro-inflammation. We report that sPIF reduces paralysis and de-myelination of the brain in a clinically-relevant experimental autoimmune encephalomyelitis mice model. These effects, at least in part, are due to post-translational modifications, which involve cyclic AMP dependent protein kinase (PKA), calcium-dependent protein kinase (PKC), and immune regulation. In terms of potential MS treatment, sPIF was successfully tested in neurodegenerative animal models of perinatal brain injury and experimental autoimmune encephalitis. Importantly, sPIF received a FDA Fast Track Approval for first in human trial in autommuninty (completed).

## Introduction

An autoimmune response against myelin protein is considered one of the key pathogenic processes that initiates multiple sclerosis (MS). In MS, trafficking of the peripherally activated myelin reactive T cells across the blood-brain barrier initiate an immune cascade leading to acute and chronic inflammation, de-myelination, and axonal injury resulting in neurodegeneration^1^. The currently available MS disease modifying therapies have demonstrated in studies to reduce the frequency of inflammatory attacks; however, they appear limited in preventing disease progression and neurodegeneration^2^. Novel therapeutic approaches addressing both inflammation and neuroregeneration are urgently needed. One approach is modulation of cyclic AMP dependent protein kinase (PKA) and calcium-dependent protein kinase (PKC) kinases^3^. PKA/PKC are important signaling molecules in a variety of cellular functions, including neuronal plasticity, cell growth and differentiation, and cellular response to hypoxia-ischemia^4,5^. Interestingly, modulation of PKC signaling leads to an unconventional communication between the immune system and nervous system with IL-4 preventing axon pathology and leading to functional recovery^6^. Given the emerging importance of PKA/PKC in neurodegenerative disorders^3^ along with synthetic PreImplantation Factor (sPIF) modulating PKA/PKC^7^, we aimed to evaluate sPIF in the experimental autoimmune encephalomyelitis (EAE) model.

PIF is a small peptide detectable in the maternal circulation during pregnancy and associated with viable embryo^8,9^. Endogenous PIF is involved with embryo implantation by modulating maternal immune tolerance^10,11^. A synthetic analog of natural PIF (sPIF) prevented paralysis and restored spinal myelination through inhibiting neuro-inflammation in murine models of EAE^12,13^. The potential of sPIF therapy for Alzheimer’s Disease was previously reported^14^ and sPIF’s ability to reduce neuronal loss and microglial activation in a murine model of perinatal brain injury provides further evidence of sPIF’s putative neuroprotective properties^7,15^. Here, we report that sPIF induces global posttranslational protein modifications in the brain, while preserving brain myelin in the EAE animal model. Further, these effects are, at least in part, due to changes of cyclic AMP dependent protein kinase (PKA) and calcium-dependent protein kinase (PKC) kinase after sPIF treatment. These effects appear in line with PIF’s pleiotropic function both in and outside pregnancy^16^ such as seen in autoimmune diabetes^17^, atherosclerosis^18^, graft versus host disease^19^, and radiation induced pathologies^20^. In terms of MS, sPIF could be useful as a treatment especially since sPIF crosses the blood-brain barrier and has successfully been tested in animal models of brain injury and inflammation^7,15^. sPIF received a FDA Fast Track Approval for first in human trial in autoimmune hepatitis.

## Results

### sPIF reverses myelination deficits

**Figure 1:**
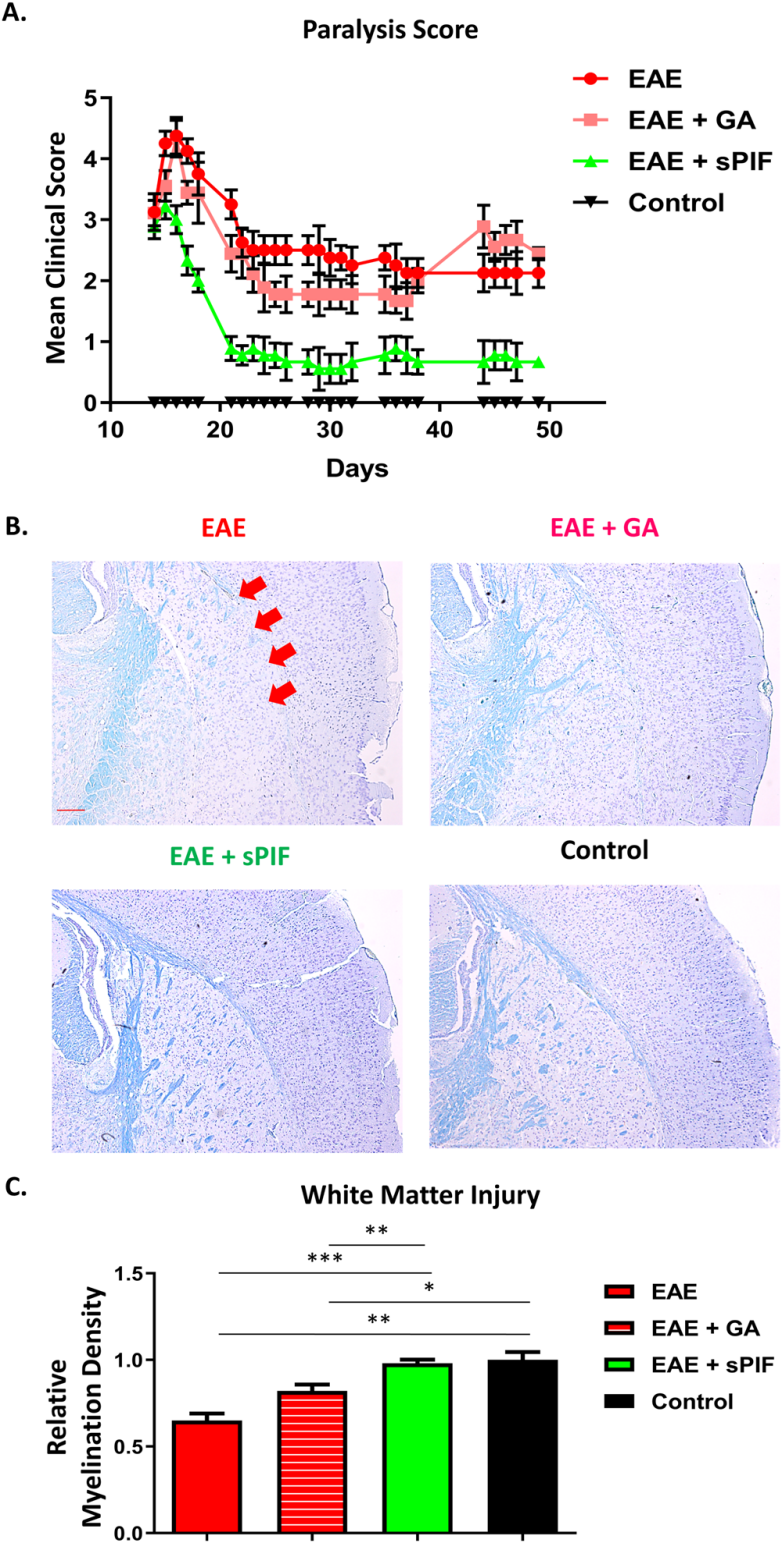
sPIF reduces clinical severity and de-myelination in EAE mice. We induced EAE in mice and started sPIF, GA, or PBS episodically once paralysis developed. (**A**) We monitored and assessed paralysis scores in mice until day 50. In Table 1 we describe detailed scores and additional time points. (**B**) At day 50 we assessed brain myelination using Luxol Fastblue staining. Representative images of subcortical white matter with de-myelination in corpus callosum (red arrows) after EAE. GA treatment restored myelination partially and sPIF completely compared to EAE and control brain. (**C**) Relative quantification of myelin density. Data presented as mean ± SEM. PBS (n = 5), sPIF (n = 6), GA (n = 5), Control (n = 3). sPIF: synthetic PreImplantation Factor, GA: glatiramer acetate, EAE: experimental autoimmune encephalomyelitis. *p < 0.05; **p < 0.01; ***p < 0.001. Non-parametric data were analyzed using the Mann-Whitney U test and mouse survival and the disease-free ratio were determined by X^2^ analysis. Scale: 200 µm.

Given that sPIF protected the spinal cord in the EAE model previously^12^ we sought to evaluate if similar neuroprotective effects occurred on the brain. We aimed to mimic the relapsing remitting MS therapy by episodic sPIF treatments. We used sPIF subcutaneously, glatiramer acetate (GA), and PBS once paralysis developed (≥score 1) and continued until paralysis regressed. In order to have a short and long-term follow up we analyzed the main outcome at three time-points. As shown in Table 1, sPIF reduced the clinical scores compared to PBS (EAE) and GA treated (EAE + GA) mice and at time point 3 sPIF was most effective (Fig. 1A and Table 1 – Time Point 3). As MS tends to be progressive leading to chronic disease of the spine and brain white matter^21^, we chose time point 3 brain tissues for further analyses. Indeed, EAE resulted in loss of myelin in the subcortical white matter tracts, which was specifically evident in the corpus callosum (Fig. 1B see red arrows). GA treatment resulted in partial re-myelination, which is in line with the modestly improved EAE clinical scores (Fig. 1B,C and Table 1). Importantly, sPIF treatment resulted in reduced brain de-myelination that closely resemble controls (Fig. 1B,C: compare green to red and black bars), which was in parallel with the observed improved clinical scores (Table 1). Together, sPIF treatment results in clinical and histological reduction of white matter loss in an EAE animal model.

**Table 1:** Clinical scores in the EAE model after GA and sPIF treatments.

		Mean Clinical Score	Peak Paralysis	Mean Clinical Score End
Time Point 1 26 days	EAE	1.36 ± 0.22	2.5 ± 0.33	1.3 ± 0.44
	EAE + GA	1.3 ± 0.15	2.4 ± 0.33	1.6 ± 0.44
	EAE + sPIF	0.95 ± 0.19	2.1 ± 0.23	*0.**60** ± **0**.**22**
Time Point 2 41 days	EAE	1.01 ± 0.19	1.0 ± 0.28	1 ± 0
	EAE + GA	0.80 ± 0.11	1.85 ± 0.23	1.4 ± 0.6
	EAE + sPIF	*0.**45** ± **0**.**08**	0.91 ± 0.14	*0.**5** ± **0**.**18**
Time Point 3 50 days	EAE	2.6 ± 0.20	4.37 ± 0.18	2.12 ± 0.85
	EAE + GA	2.4 ± 0.15	4.33 ± 0.16	2.44 ± 0.53
	EAE + sPIF	*1.**2** ± **0**.**10**	*3 ± **0**.**33**	*0.**66** ± **0**.**55**

Clinical score analysis of sPIF and GA at the three different time points in EAE mice. We ended the experiment at Time Point 1: 26 days, Time Point 2: 41 days, and Time Point 3: 50 days. For further analyses we selected Time Point 3 as the effect of sPIF was profound. MCS = mean clinical score, PP = peak paralysis, MCE = mean clinical score end. sPIF: synthetic PreImplantation Factor; GA: glatiramer acetate. EAE: experimental autoimmune encephalomyelitis. Data is expressed as mean +/− SEM. sPIF, GA and PBS: n = 7–18 per group. *p < 0.05 compared to EAE. Control (healthy animals) are not presented as the score was 0.

### sPIF induces global phosphorylation changes in the brain

**Figure 2:**
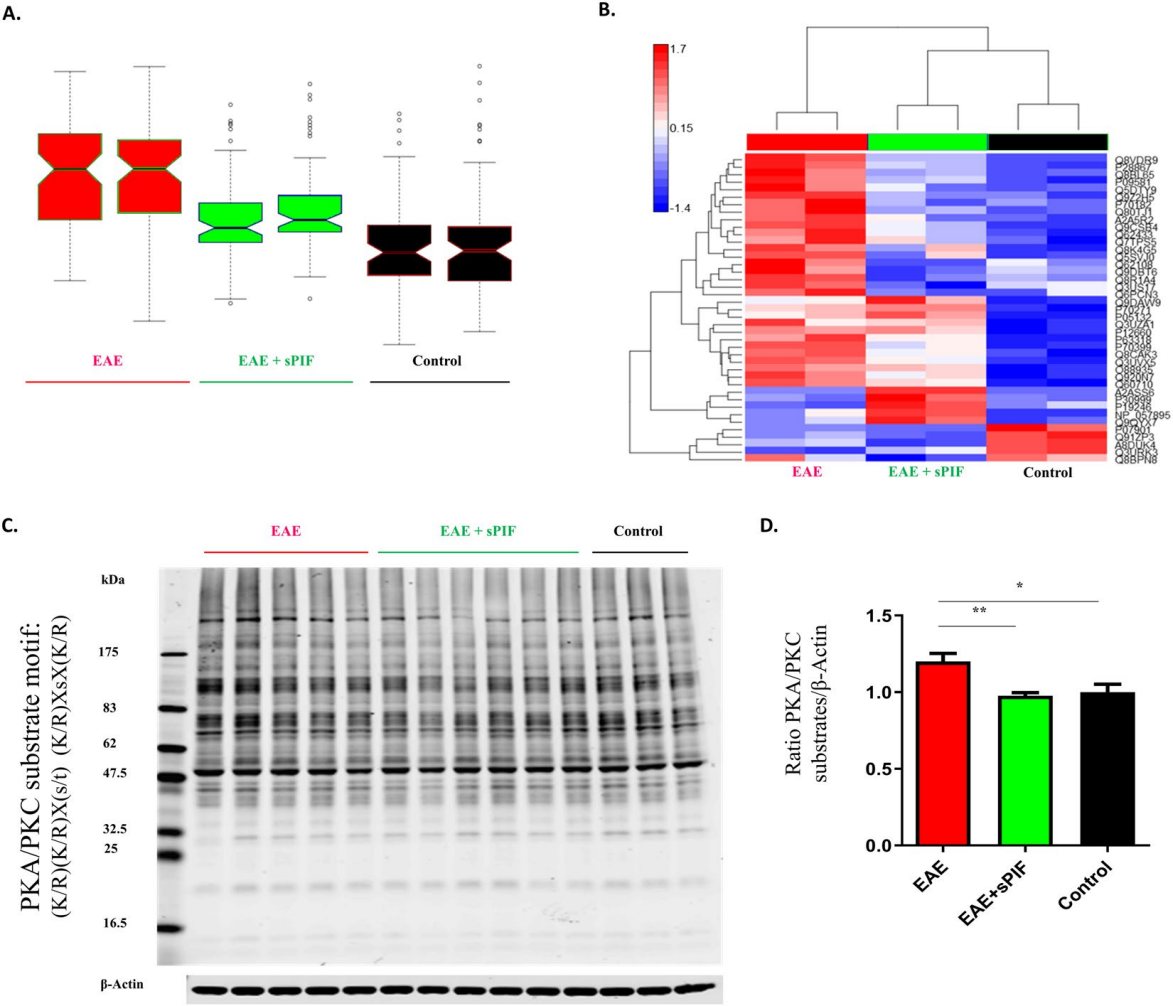
sPIF induces post-translational modifications in the EAE brains. (**A**) Box-and-Whisker graph of global normalized abundance and significantly expressed phosphoproteins. (**B**) The heat map shows clustered protein expression (rows) using unsupervised hierarchical clustering of phosphoproteins. Intensity ranges from highest intensity (red) to lowest (blue). Detailed protein list is presented in Tables 2 and 3. (**C**) Representative western blots of brain lysates probed with antibodies against specific (K/R)(K/R)X(s/t) and (K/R)XsX(K/R) phosphorylation motifs representing PKA/PKC substrates. β-Actin was used as a loading control. (**D**) Quantitative assessment of PKA/PKC kinase modulated proteins levels were quantified using beta-actin as a loading control. sPIF: synthetic PreImplantation Factor, EAE: experimental autoimmune encephalomyelitis. *p < 0.05; **p < 0.01; Data are presented as mean ± S.E.M. (one-way repeated measures ANOVA followed by Bonferroni’s Multiple Comparison Test, two-tailed Student’s t-test).

**Figure 3:**
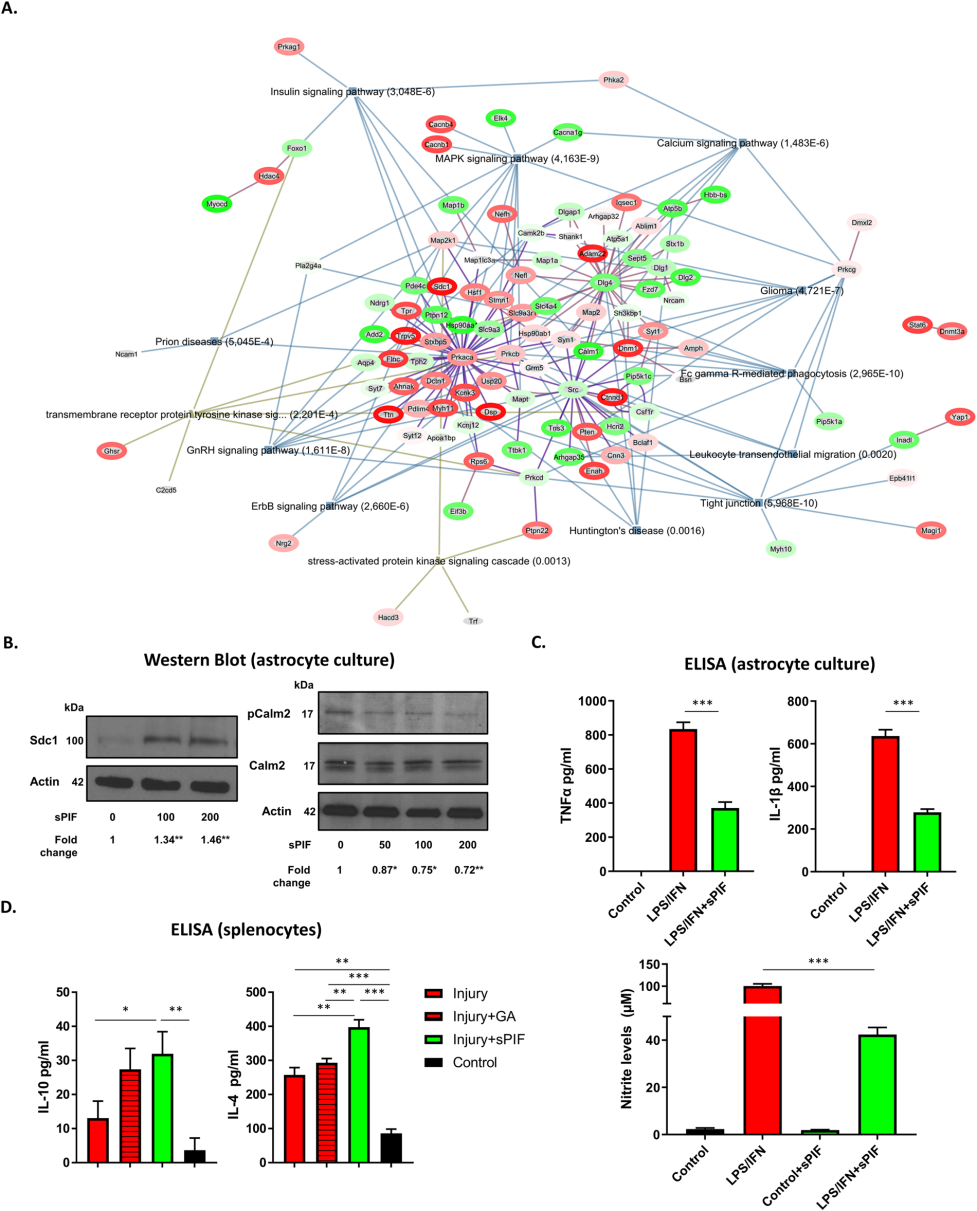
sPIF modulates PKA/PKC signaling and inflammation. (**A**) Gene enrichment and network linkage analysis hypergraph of phosphoproteins created using EGAN. The hypergraph is annotated with signaling pathways and GO Process terms. Kinase-substrate interaction is encoded as violet edges. Enrichment probability is depicted by the thickness of borders, upregulation is colored red and downregulation is colored green. Data is represented as fold change of differentially expressed proteins in sPIF compared to EAE mice treated with PBS. (**B**,**C**) Validation of the global analysis using western blots. Cultured astrocytes were treated with sPIF (or control) in dose dependent manner. Representative western blots showing decreased phosphorylation of Calm2 and increased expression of Sdc1. (**C**) Cultured astrocytes were challenged with LPS/IFN and treated with sPIF or control. Using Elisa we detected reduced proinflammatory cytokines TNFα and IL-1β and decreased NO production after sPIF treatment. (**D**) EAE mice derived splenocytes were activated and supernatant tested for cytokines using ELISA. sPIF increases IL-10 and IL-4 levels. sPIF: synthetic PreImplantation Factor, GA: glatiramer acetate, EAE: experimental autoimmune encephalomyelitis. LPS: Lipopolysaccharides, IFN: Interferon γ. *p < 0.05; **p < 0.01; ***p < 0.001. Data are presented as mean ± S.E.M. (one-way repeated measures ANOVA followed by Bonferroni’s Multiple Comparison Test, two-tailed Student’s t-test).

To better understand the sPIF-induced changes (Fig. 1), we selected a global screening approach using antibody-based enrichment from well-characterized post-translational modifications (PTMs)^7,22^. These antibodies detect targeted PTMs in the context of specific binding motif or substrate and therefore present a global overview of induced changes. In order to identify specific changes, we combined the antibody-based immunoprecipitation PTMs enrichment with liquid chromatography-tandem mass spectrometry (Orbitrap LQ-MS). We excluded GA-treated EAE mice, as the treatment was not superior to sPIF (Table 1 and Fig. 1). As seen in the Whisker box plots (Fig. 2A) EAE resulted in an increased global (normalized) abundance of significantly expressed phosphoproteins and with sPIF treatment this abundance was decreased. We confirmed these observations using unsupervised hierarchical clustering of phosphoproteins (Fig. 2B – heat map). Notably, we aimed to cover highly diverse regulatory pathways using a novel combination of phospho-Ser/Thr motif antibodies to provide broader coverage of the phosphoproteome^23^. Using this screening approach, we detected changes of cyclic AMP dependent protein kinase (PKA) and calcium-dependent protein kinase (PKC) kinase after sPIF treatment (Fig. 2C,D). Notably, PKA/PKC are important signaling molecules in a variety of cellular functions, including neuronal plasticity or cell growth and differentiation^4,5^. Modulation of PKA/PKC in MS was previously reported^1,3^. The detailed analysis and clustering of the significantly modulated proteins is summarized in Tables 2 and 3 and as expected, both EAE and sPIF modulated multiple phosphoproteins. To put the induced interactions into context, we used PhosphoSitePlus derived Kinase-Substrate data in combination with gene-enrichment and network linkage analysis of phosphoprotein expression data (Fig. 3A). We detected multiple changes and cAMP-dependent protein kinases, with catalytic subunit alpha (Prkaca), along with Src and its substrate Dlg4, take a central role. Additionally, the most abundant changes induced by sPIF were Dsp (desmoplakin; reduction ~50-fold), followed by Sdc1 (syndecan-1; reduction ~38-fold), and Calm2 (Calmodulin2; increase ~5-fold) as seen in Table 3. Notably, these proteins are associated with neurodegenerative and neuroinflammatory diseases^24–26^.

**Table 2:** Clustering analysis of phosphoprotein differential expression.

ID	Gene Name
**Phosphoproteins significantly reduced by sPIF but not by EAE**	
*Arfgef2*	ADP-ribosylation factor guanine nucleotide-exchange factor 2 (brefeldin A-inhibited)
*Cadps*	Ca^2+^ -dependent secretion activator
*Ndrg1*	N-myc downstream regulated gene 1
*Ablim1*	actin-binding LIM protein 1
*Ablim2*	actin-binding LIM protein 2
*Camk2b*	calcium/calmodulin-dependent protein kinase II, beta
*Csf1r*	colony stimulating factor 1 receptor
*Dock7*	dedicator of cytokinesis 7
*Dlg4*	discs, large homolog 4 (Drosophila)
*Pard3b*	par-3 partitioning defective 3 homolog B (*C*. *elegans*)
*Pip5k1a*	phosphatidylinositol-4-phosphate 5-kinase, type 1 alpha
*Kctd16*	potassium channel tetramerisation domain containing 16
*Prkcd*	protein kinase C, delta
*Ttbk1*	tau tubulin kinase 1
*Tns1*	tensin 1
*Pdlim4*	PDZ and LIM domain 4
*Pcp2*	Purkinje cell protein 2 (L7)
*Rcsd1*	RCSD domain containing 1
*Grm5*	glutamate receptor, metabotropic 5
*Samhd1*	hypothetical protein LOC100045969; SAM domain and HD domain, 1
*Prkaca*	protein kinase, cAMP dependent, catalytic, alpha
*Cnn3*	similar to calponin 3, acidic; predicted gene 4815; calponin 3, acidic
*Syn1*	synapsin I
*Syt12*	synaptotagmin XII
**Phosphoproteins significantly induced by sPIF but not by EAE**	
*Ctnnd1*	catenin (cadherin associated protein), delta 1
*Pclo*	piccolo (presynaptic cytomatrix protein); hypothetical protein LOC100044163
*Nefh*	similar to neurofilament protein; neurofilament, heavy polypeptide
*Ttn*	Titin
*Vezf1*	vascular endothelial zinc finger 1
**Phosphoproteins significantly induced by EAE but not by sPIF**	
*Dmxl2*	Dmx-like 2
*Lpin1*	lipin 1
*Hsp90aa1*	heat shock protein 90, alpha (cytosolic), class A member 1
*Tet1*	tet oncogene 1

Detailed analysis of phosphoproteins in the brain induced after EAE and/or treatments at Time Point 3 (50 days). sPIF: synthetic PreImplantation Factor; EAE: experimental autoimmune encephalomyelitis.

**Table 3:** Phosphoprotein expression classified by EGAN probability analysis.

Entrez Gene	Canonical Name	EAE vs Control	EAE + sPIF vs EAE	EAE + sPIF vs Control
109620	Dsp	1.8582592	**48**.**9470834**	**90**.**8832658**
22138	Ttn	0.993652	**45**.**4578046**	**87**.**1599315**
20969	Sdc1	1.6684294	**37**.**2113239**	**62**.**0345661**
16678	Krt1	2.2114833	24.3724923	53.8560382
223650	Eppk1	4.7528523	6.1181544	29.0553129
100177	Zmym6	−1.002286	20.2999401	20.2373644
71752	Gtf3c2	3.7822468	5.24402904	19.8182708
224432	Scaf4	**112**.**08234**	−7.9380282	14.1083223
68760	Synpo2l	−1.200038	16.5643611	13.7921068
118449	Synpo2	1.4249756	8.80237652	12.5330903
70233	Cd2bp2	**122**.**52817**	−9.8414457	12.440214
105245	Txndc5	**176**.**12964**	−16.722331	10.5241357
59006	Myoz2	1.3498843	6.8535988	9.24412972
217615	Ctage5	2.1995328	3.34714634	7.356241
194352	Trpv5	−1.137748	7.85847523	6.90149395
67228	Dph7	−1.487115	9.50958865	6.38951462
226594	Rcsd1	5.4588784	1.06107557	5.78762707
16527	Kcnk3	2.0452615	2.78275384	5.68688489
140780	Bmp2k	2.6330244	2.10780982	5.54545416
26386	Hsf4	3.250466	1.68234731	5.46401758
215476	Prr14l	−1.865828	−2.8858887	−5.3889041
11947	Atp5b	−2.215433	−2.6252907	−5.8208343
268373	Ppia	−5.674568	−1.1228766	−6.3769652
18039	Nefl	−15.53543	1.70047011	−9.1433112
19153	Prx	−4.408617	−2.5637779	−11.311805
100503605	Hbb-bs	−3.799876	−3.110475	−12.411454
12313	Calm2	−6.542107	−4.6028786	−30.136746
15519	Hsp90aa1	−5.915526	−6.8662554	−46.925216

Detailed analysis of phosphoproteins in the brain induced after EAE and/or treatments at Time Point 3 (50 days). We ranked the expression by sPIF effect versus control mice and only proteins that showed a 5-fold increase or decrease (or more) are listed. The positive numbers indicate increase (three highest are bold) and negative numbers decrease of phosphoprotein expression. sPIF: synthetic PreImplantation Factor; EAE: experimental autoimmune encephalomyelitis.

### sPIF modulates inflammation and astrocytes

To validate the global phosphorylation changes induced by sPIF, we chose Sdc1 and Calm2. Notably, Sdc1 is a heparin sulfate fibroblast growth factor with anti-inflammatory properties^27^. Sdc1 knock-down results in enhanced EAE severity and impaired recovery due to increased immune cell recruitment to the brain^27^. The second chosen protein was Calm2. It is a member of calcium signaling, which is important during brain injury and inflammation^26^. In order to confirm the globally detected effects in the brain (Fig. 3A, Tables 2 and 3), we cultured astrocytes as they contribute to neuroinflammation in EAE^28^ and used sPIF treatment in increasing concentration. Additionally, we chose astrocytes for *in-vitro* testing as the astrocytes in white matter are associated with inflammation that is PKA-dependent^29^. In line with our global screening approach (Fig. 3A), sPIF increased Sdc1 expression and reduced Calm2 phosphorylation (Fig. 3B) confirming the detected global changes (Tables 2 and 3). Further, we performed an inflammatory challenge of the primary astrocytes and used sPIF treatment. In line with previous result^12,19,20^, sPIF reduced the proinflammatory cytokines TNFα and IL-1β while decreasing the NO production (Fig. 3C). To further confirm sPIF’s anti-inflammatory effect, we tested EAE mice splenocytes cultured with proteolipid protein (PLP) for re-activation. Again, we chose the splenocytes as these represent a communication between the immune system and nervous system^6^ and sPIF suppressed the proinflammatory cytokines IL17 and IL6 after PLP-stimulated EAE derived splenocytes previously^12^. Here we demonstrate that sPIF increases both IL-10 and IL-4 anti-inflammatory cytokines secretion (Fig. 3D)^12,13^.

Together, sPIF reduced the magnitude of de-myelination and helped improve clinical scores in a clinically relevant MS animal model. These effects, at least in part, were due to global post-translational modification of phosphoproteins, which involved PKA/PKC signaling in combination with immune control.

## Discussion

Neuroinflammation, especially inflammation leading to MS development, is a common cause of neurological disability^16^. Although, newer MS disease modifying therapies appear to reduce the magnitude of the inflammatory phase of the disease, disease progression and neurodegeneration is minimally impacted. We demonstrated in this study sPIF’s potential to address this gap in treatment, which in part is by post-translational modification of phosphoproteins that are involved in PKA/PKC signaling. The specific effect on PKA/PKC signaling is intriguing as the effect (Fig. 2C,D) is partially divergent from previous report^7^. However, the spatio-temporal dynamics on multiple PKA/PKC subunits and the effect of a developing brain need to be accounted for^30^. Further, we validated the global phosphorylation changes (Fig. 3A, Tables 2 and 3) using Sdc1 and Calm2 in astrocytes. We chose those proteins due to their involvement in inflammation in the brain^26,27^. We chose astrocytes for *in-vitro* testing as astrocytes are contributing to neuroinflammation in MS and EAE model^28,29^. Finally, the PKA/PKC modulation as a therapeutic approach for other neurodegenerative diseases such as cerebral ischemic/stroke and Alzheimer’s Disease was previously demonstrated^31–33^. Therefore, Alzheimer’s Disease, perinatal brain injury, and MS are potential disease candidates for successful sPIF treatment^7,12–15^. Other diseases such as anti N-methyl-D-aspartate ion channel receptor (NMDAR) autoimmune encephalitis are potential candidates as well. We identified Dlg4 and calcium/calmodulin-dependent protein kinase members (Table 2 and Fig. 3A) as PIF targets after EAE and these regulate NMDAR activity^34,35^. Together, although current evidence is derived from animal models, sPIF is a promising therapeutic agent to treat multiple neurodegenerative disorders especially since sPIF crosses the blood-brain-barrier and received a FDA Fast Track Approval for first in human trial in autoimmune hepatitis, which was successfully completed^36^.

We acknowledge that there are several limitations of this study such as the observational character of our study or the animal model itself. As most animal models of MS, the EAE model does not directly represent the human disease. However, the EAE model led to introduction of other MS drugs and is suitable to study MS pathogenesis and candidate therapies^37^. The EAE animal model results in T-cell mediated activation and differentiation into encephalitogenic Th1/Th17 cells^38^. Auto-reactive Th1 and Th17 cells and antigen presenting cells play an essential role in MS pathogenesis and not surprisingly IL-6 or TGF-β cytokines are detected in chronic MS brain lesions^39^. The sPIF-induced increase in IL-10 and IL-4 secretion (Fig. 3D) is in line with the decrease of pro-inflammatory cytokines in chronic EAE and Mycobacterium Smegmatis induced neuroinflammation models^12,13^. Interestingly, intrathecal and intranasal IL-4 treatment during the chronic phase of several EAE models reversed disease progression^6^. The question whether sPIF will be effective in viral models reflecting key features of MS-like inflammatory de-myelination is still open but beyond the scope of this manuscript. Additionally, the exact pathways modulated by sPIF in EAE needs further attention, although, this study offers important insights that could help guide further studies. sPIF has the potential to impact the inflammatory aspect(s) of MS in part by modulating post-translational modification of phosphoproteins and has a putative re-myelinating properties. Therefore, sPIF is a strong candidate for human clinical trials in MS.

## Materials and Methods

### Animal studies

#### Mice

SJL mice (5–6 weeks old female) were obtained from Harlan Laboratories Ltd. (Israel). Mice were kept and monitored in SPF conditions with autoclaved cages. The study was conducted under appropriate conditions and approved by the Institutional Animal Welfare Committee of the Hebrew University of Jerusalem. All methods were performed in accordance with the relevant guidelines and regulations. We induced EAE in mice as previously described^12^. Briefly, SJL females (N = 7–18/group in four independent experiments) were immunized subcutaneously on day 0 with a 1:1 emulsion comprised of 100 μg proteolipid protein (PLP) (aa 139–151 peptide) and *Mycobacterium tuberculosis* H37R (BD Biosciences) in complete Freund’s adjuvant (CFA) to final volume of 2 mg/ml. At day 0 and day +2, pertussis toxin (Sigma Chemicals, St. Louis, MO) was administered (250 ng/mouse) intra-peritoneal (I.P.) injection. Mice were monitored daily, starting on day +6 until sacrifice.

sPIF (0.75 mg/kg bodyweight subcutaneously), Glatiramer acetate (GA) (5 mg/kg bodyweight) or PBS was administered twice daily. We initiated treatment once paralysis was documented defined as score 1 and/or above. We continued the treatment until paralysis totally regressed (0). The treatment was started episodically again only when a given mouse had started to develop signs of paralysis and stopped when paralysis totally resolved.

#### PIF synthesis

Synthetic PIF (sPIF), proprietary fifteen-amino-acid peptide (MVRIKPGSANKPSDD), was produced using solid-phase peptide synthesis (Peptide Synthesizer, Applied Biosystems, Foster City, CA) employing Fmoc (9-fluorenylmethoxycarbonyl) chemistry^12^. Final purification was carried out by reversed-phase high-pressure liquid chromatography (HPLC), peptide identity was verified by matrix-assisted laser desorption/ionization time-of-flight (MALDI-TOF) mass spectrometry and amino acid analysis, and the peptide was purified to >95% by HPLC, as documented by mass spectrometry was generated (Polypeptide Labs, CA). Clinical grade Glatiramer acetate (GA) (Teva, Israel) was received as a gift from Dr. Karousis, Hadassah Medical Center, Department of Neurology.

#### Clinical evaluation of neuroinflammation

The separation of mice to groups was randomized^12^. The first signs of paralysis appeared within a few days post-immunization which varied due to the intensity of the disease. In general, the clinical signs started within 6–11 days (~9) from inoculation. Mice were monitored daily and scored using the standard EAE six-point scale: 0–normal behavior; 1-low tail tonus; 2-hind-leg weakness; 3-hind-leg paralysis; 4-full paralysis; and 5-death^12^. In all cases, we calculated the following scores: (1) mean clinical score (MCS) defined as the average of the daily scores of all mice within each group. (2) mean peak paralysis scores (PP) defined as the average of individual scores of all mice in the group. (3) mean clinical score at end (MCE) defined as the average score of all mice within each group on the last day of study (day 26–50 of the experiment).

#### Splenocytes cultures and cytokines analysis

Method of culture and cytokine testing were previously reported^12^. Briefly, spleens from sacrificed mice from sPIF, GA, PBS-treated or naïve mice groups were harvested and their splenocytes isolated. Cells were cultured in DMEM medium supplemented with 10% fetal bovine serum, 2 mM L-glutamine, 100 U/ml penicillin and 100 μg/ml streptomycin. Cells were cultured in duplicate at 5 × 10^6^ cells/well in presence of 4 mg/ml PLP peptides. Negative control wells were cultured without PLP peptides, whereas positive control wells were cultured with 2.5 μg/ml concanavalin A (ConA). After 3 days of incubation, the supernatant was collected and the cytokine levels were determined by using FlowCytomix Mouse Th1/Th2 10plex kit according to the manufacturer recommendation (eBioscience).

#### Astrocyte cultures and measurment of inflammatory markers

Mixed astrocyte cultures were obtained as previously performed^13^. Briefly, cultures were prepared from 1–2 day old C57BL/6 mouse neonates with cortices isolated, cut into small pieces, and incubated with 2.5% Trypsin for 30 min at 37 °C. Following centrifugation, the tissue pellet was mechanically dissociated into single cells. Cells were plated in DMEM, high glucose +10% heat-inactivated fetal bovine serum +1% Penicillin/Streptomycin. After 8 days, confluent cultures undergo a series of shaking to remove microglia and oligodendrocyte precursor cells and the astrocytes are trypsinized and replated. Purity of the primary astrocytes was determined by immunofluorescence staining with GFAP and GLAST (Over 95% positive cells). The inflammatory challenge was performed using LPS (10 µg/ml) and IFN-g (3 ng/ml) with or without sPIF (100 nM) for 24 hours. The secretion of TNF-α and IL-1β was determined in tissue supernatants using ELISA kits (Abcam: ab100747 and ab100705) according to manufacturer’s instruction. The production of NO by astrocytes was analyzed by indirect measurement of nitrite concentration using the colorimetric Griess assay (Sigma-Aldrich G4410) according to manufacturer’s instruction. A standard curve of sodium nitrite was used for calibration.

#### Histological analysis

Brain tissue samples (sPIF, GA, PBS and naïve mice) were fixed in 4% neutral-buffered formalin, embedded in paraffin and sectioned into 7 μm slices. Slides were rinsed in DDW, counterstained in Cresyl violet and Luxol Fast Blue, dehydrated in ethanol and xylene, and mounted with Eukitt (Sigma-Aldrich, St. Louis, MO)^13^. We acquired images using a BX51 microscope (Olympus, Tokyo, Japan) with a 20× or 40× objective and equipped with a digital camera. An independent observer acquired sections visual field by visual field without overlapping per hemisphere and animal for each specific staining blinded to the experimental conditions. The region of interest (ROI) was the corpus callosum^13^. To asses myelin loss (MBP immunostaining) we measured the percentage of positive MBP staining (area) in the ROI as described previously^22,40^.

#### Tissue lysate preparation and phosphoproteome analysis

PTMScan phosphorylation analysis was performed on mouse brain samples as previously described^23^. Mice brain tissue (PBS n = 5, sPIF n = 6, naïve untreated control n = 3) obtained from the chronic EAE experiment on day 50, was deeply frozen. An aliquot of brain tissue extracts was removed for protein concentration determination with BCA assay (Thermo Fischer). We placed 20 mg of mouse tissue extract per condition in 5 ml urea lysis buffer reduced with 4.5 mM DTT for 30 min at 55 °C, alkylated with iodoacetimide (0.095 g per 5 mL H_2_O) for 15 min at room temperature in the dark. We diluted samples 1:4 with 20 mM HEPES pH 8.0 and digested overnight with 50 µg LysC (Wako, #129–02541) per 5 mg cellular protein. We normalized soluble protein amounts for each sample prior to digestion to ensure equal protein input for all samples. We acidified the digested peptide lysates with 1% TFA and desalted the peptides over 360 mg SEP PAK Classic C18 columns (Waters, #WAT051910). We eluted the peptides with 40% acetonitrile in 0.1% TFA, dried under vacuum, and stored at −80 °C. For Phosphoprotein Immunoprecipitation we used following setup as previously performed {Mueller, 2015 #17}. PTMScan motif antibodies: Akt (#9614 & #10001), PKA (#9624), PKC (#2261), PKD (#4381), CDK (#2324), AMPK (#5759), ATM/ATR (#6966 & #9607), CK (#8738), PDK1 (#9634), MAPK (#2325), tP (#8134), tPE (#3004), PLK (#5243), tXR (#8139), 14-3-3 (#9442) were combined and conjugated to 40 ul of Protein A agarose (Roche) overnight at 4 °C. Lyophilized peptides were re-suspended in MOPS IAP buffer (50 mM MOPS pH 7.2, 10 mM KH_2_PO_4_, 50 mM NaCl) and centrifuged 5 min at 12,000 RPM in a MiniSpin microcentrifuge (Eppendorf). Supernatants were mixed with PTMScan Reagent-Bead slurries 2 hours at 4 °C. Beads were pelleted by centrifugation 30 seconds at 5,400 RPM in a MiniSpin microcentrifuge at 4 °C. Beads were washed twice with 1 mL MOPS IAP buffer and four times with 1 mL water (Burdick and Jackson). Peptides were eluted from beads with 0.15% TFA (sequential elutions of 65 µL followed by 55 µL, 10 min each at room temperature). Eluted peptides were desalted over tips packed with Empore C18 (Sigma) and eluted with 40% acetonitrile in 0.1% TFA. Eluted peptides were dried under vacuum. LysC digested peptides were subjected to a second, in-solution trypsin digest using 250 ng of sequencing grade trypsin (Promega) in 50 mM ammonium bicarbonate/5% acetonitrile for 2 hours at 37 °C. Samples were acidified with TFA and re-purified over C18 tips as before. We used following MS Parameter Settings**:** MS Run Time 90 min, MS1 Scan Range (300.0–1500.00), Top 20 MS/MS (Min Signal 500, Isolation Width 2.0, Normalized Coll. Energy 35.0, Activation-Q 0.250, Activation Time 20.0, Lock Mass 371.101237, Charge State Rejection Enabled, Charge State 1+ Rejected, Dynamic Exclusion Enabled, Repeat Count 1, Repeat Duration 35.0, Exclusion List Size 500, Exclusion Duration 40.0, Exclusion Mass Width Relative to Mass, Exclusion Mass Width 10ppm). Real time recalibration of mass error was performed using lock mass with a singly charged polysiloxane ion m/z = 371.101237.

#### LC-MS/MS analysis

We re-suspended the immunoprecipitated peptides in 0.125% formic acid and separated them on a 10 cm × 75 μm PicoFrit capillary column packed with Magic C18 AQ reversed-phase resin. The column was developed with a 120-minute linear gradient of acetonitrile in 0.125% formic acid delivered at 280 nL/min. Each sample was split. We run analytical replicate injections to increase the number of identifications and additionally to provide metrics for analytical reproducibility. Replicate injections were run non-sequentially to reduce artifact-associated changes in peptide abundance due to changes in instrument performance over time. One replicate of each sample was injected, then the second replicate in reverse order. Tandem mass spectra were collected in a data-dependent manner with an LTQ-Orbitrap Velos mass spectrometer running XCalibur 2.0.7 SP1 using a top-twenty MS/MS method, a dynamic repeat count of one, and a repeat duration of 30 sec. We used following LC-MS^2^ PRIMARY Data Analysis**:** MS/MS spectra were evaluated using SEQUEST, and the Core platform from Harvard University^23^. Files were searched against the NCBI *Mus musculus* FASTA database, release date 06/28/2011. A mass accuracy of +/−50 ppm was used for precursor ions and 1 Da for product ions. We limited the enzyme specificity to LysC/trypsin, with at least one LysC or tryptic (K- or R-containing) terminus required per peptide and up to four mis-cleavages allowed. Cysteine carboxamidomethylation was specified as a static modification. Oxidation of methionine residues was allowed. Phosphorylation was allowed on serine, threonine, and tyrosine residues. We included reverse decoy databases for all searches to estimate false discovery rates and filtered using a 1% FDR in the Linear Discriminant module of Core. We further narrowed the results by mass accuracy based on clustering of forward and reverse assignments in Xcorr versus mass error plots. Typically, forward database assignments cluster within −/+5 ppm of calculated m/z, so results were limited to peptides that fall within that range. We used a larger mass error range (−/+50 ppm) for the searches to allow for identification even if the lock mass signal was not adequate for accurate mass calibration. We manually filtered peptides using reagent-specific criteria. For each antibody reagent results were filtered to include only phosphoproteins matching the sequence motif(s) targeted by the antibodies included. All quantitative results were generated using Skyline Version 3.1 to extract the integrated peak area of the corresponding peptide assignments. Extracted ion chromatograms for peptide ions that changed in abundance between samples were manually reviewed to ensure accurate quantitation in Skyline 3.1 or XCalibur software (version 2.0.7 SP1, Thermo Scientific). We normalized peak areas using a log2 median normalization strategy as previously described^23^. To identify proteins with statistically significant changes in experimental groups (PBS treated EAE mice, sPIF treated EAE mice and intact mice), we used the label-free quantification (LFQ) intensities. LFQ values were normalized and ratios between EAE mice and control mice, sPIF treated EAE mice and EAE mice, as well as sPIF treated EAE mice to control estimated.

#### Differential phosphoprotein expression analysis

Peptide abundances among technical replicates were combined, log_2_-transformed, normalized and converted to protein abundances with the ZRollup method implemented in InfernoRDN (http://omics.pnl.gov/software/infernordn). Only proteins with two or more unique peptides were retained for ANOVA analysis (P-values < 0.05 and q-values to control the false discovery rate below 0.04 in multiple testing^41^. Unsupervised hierarchical clustering was used for grouping both individual experimental samples and ZRollup estimated protein abundances. Heatmap with top (samples) and left (proteins) dendrogram was produced (Fig. 3B). Proteins were clustered using Agglomeration Average method applying Euclidean distance metric, while samples were clustered using Complete Linkage method applying Euclidean distance metric. We further used EGAN: Exploratory Gene Association Networks (http://akt.ucsf.edu/EGAN/) on ANOVA filtered proteins for hypergraph visualization of proteins and meta-data enrichment analysis (Gene Ontology annotation, KEGG, PANTHER signaling pathways). Protein expression fold change between treatment groups were calculated and fed to EGAN. Additionally, an extra Kinase-substrate interaction node linking file was produced, using information from PhoshpositePlus database (http://www.phosphosite.org/, CellSignaling, MA, US). The Analysis of phosphosite correlation to kinase/phosphatase and phosphor-peptide associations was used. Peptide and protein expression was analyzed SELPH platform (*Systematic Extraction of Linked Phospho-Interactions*) for correlation analysis of phospho-sites to extract kinase/phosphatase and phospho-peptide associations and link this data to GO and pathway enrichment^42^.

#### Verification studies

For verifications of the LC-MS/MS results we performed Western blot analysis in astrocytes. Mouse brain mixed astrocytes were isolated and were treated with sPIF at given concentration for 24 hours. The expression of Syndecan-1 and CALM2 was determined using Western blot analysis. Rabbit anti-syndecan 1 (Abcam: ab34164, USA) was used at a dilution of 1:3000 and mouse anti-CALM2 (Abcam: ab2860, USA) and phospho anti-CALM2 (Abcam: ab61001, USA), and actin (Abcam: ab8227, USA) were used at a dilution of 1:1000. Western blot analysis of the protein samples was performed as previously described^7^. Briefly, 20–30 µg aliquots of total protein were electrophoresed, transferred onto PVDF membrane and probed with the specific antibodies. Detection of HRP-conjugated antibodies was performed using SuperSignal (Pierce, Rockford, IL). Equal protein loading was examined using Ponceau S staining or probing the membrane with anti-actin antibody.

For analysis of the individual phospho-peptide sequences, phosphorylation sites, peptide abundances according to mice treatment scenarios, corresponding protein PPIs, we carried out Western Blotting as well. Protein concentrations were measured by Bradford Assay and 25 µg of total protein was run in each lane for western blotting. Western blots were developed using a LI-COR Odyssey NIR (near infrared) imaging system. Normalized quantitation was performed to compare relative abundance of individually detected proteins in EAE model, sPIF treated EAE model and intact (PBS-treated) scenarios.

#### Statistical analysis

Non-parametric data were analyzed using the Mann-Whitney U test. Mouse survival and the disease-free ratio at the end of the study were determined by X^2^ analysis. P < 0.05 was considered statistically significant. For other analyses comparison among the treated groups were carried initially using ANOVA followed by t test to determine significance set at p < 0.05.
